# Mesial-Temporal Epileptic Ripples Correlate With Verbal Memory Impairment

**DOI:** 10.3389/fneur.2022.876024

**Published:** 2022-06-03

**Authors:** Jonas Christian Bruder, Kathrin Wagner, Daniel Lachner-Piza, Kerstin Alexandra Klotz, Andreas Schulze-Bonhage, Julia Jacobs

**Affiliations:** ^1^Clinic of Neuropediatrics and Muscle Disorders, Freiburg University Medical Center, Breisgau, Germany; ^2^Abteilung Epileptologie Epilepsiezentrum, Klinik Für Neurochirurgie, Universitätsklinikum Freiburg, Breisgau, Germany

**Keywords:** epilepsy, temporal lobe epilepsy, high frequency oscillations (HFO), ripples, memory, sleep spindles

## Abstract

**Rationale:**

High frequency oscillations (HFO; ripples = 80–200, fast ripples 200–500 Hz) are promising epileptic biomarkers in patients with epilepsy. However, especially in temporal epilepsies differentiation of epileptic and physiological HFO activity still remains a challenge. Physiological sleep-spindle-ripple formations are known to play a role in slow-wave-sleep memory consolidation. This study aimed to find out if higher rates of mesial-temporal spindle-ripples correlate with good memory performance in epilepsy patients and if surgical removal of spindle-ripple-generating brain tissue correlates with a decline in memory performance. In contrast, we hypothesized that higher rates of overall ripples or ripples associated with interictal epileptic spikes correlate with poor memory performance.

**Methods:**

Patients with epilepsy implanted with electrodes in mesial-temporal structures, neuropsychological memory testing and subsequent epilepsy surgery were included. Ripples and epileptic spikes were automatically detected in intracranial EEG and sleep-spindles in scalp EEG. The coupling of ripples to spindles was automatically analyzed. Mesial-temporal spindle-ripple rates in the speech-dominant-hemisphere (left in all patients) were correlated with verbal memory test results, whereas ripple rates in the non-speech-dominant hemisphere were correlated with non-verbal memory test performance, using Spearman correlation).

**Results:**

Intracranial EEG and memory test results from 25 patients could be included. All ripple rates were significantly higher in seizure onset zone channels (*p* < 0.001). Patients with pre-surgical verbal memory impairment had significantly higher overall ripple rates in left mesial-temporal channels than patients with intact verbal memory (Mann–Whitney-U-Test: *p* = 0.039). Spearman correlations showed highly significant negative correlations of the pre-surgical verbal memory performance with left mesial-temporal spike associated ripples (r_s_ = −0.458; *p* = 0.007) and overall ripples (r_s_ = −0.475; *p* = 0.006). All three ripple types in right-sided mesial-temporal channels did not correlate with pre-surgical nonverbal memory. No correlation for the difference between post- and pre-surgical memory and pre-surgical spindle-ripple rates was seen in patients with left-sided temporal or mesial-temporal surgery.

**Discussion:**

This study fails to establish a clear link between memory performance and spindle ripples. This highly suggests that spindle-ripples are only a small portion of physiological ripples contributing to memory performance. More importantly, this study indicates that spindle-ripples do not necessarily compromise the predictive value of ripples in patients with temporal epilepsy. The majority of ripples were clearly linked to areas with poor memory function.

## Introduction

Despite optimized medical treatment approximately one-third of all patients with epilepsy continue to suffer from epileptic seizures (1). Among these intractable epilepsies, temporal lobe epilepsy is the most common cause. In total, 70% of cases with mesial-temporal lobe epilepsy (mTLE) are associated with mesial-temporal sclerosis (2, 3). Surgical removal of epileptic tissue is a curative option for refractory epilepsy. In temporal lobe epilepsies, selective amygdalohippocampectomy (sAHE) and anterior temporal lobe resection (ATL) are the main evidence-based surgical options (4). In order to perform successful surgery, epileptologists have to define the seizure onset zone (SOZ) in patients, which is defined as the brain area that generate seizures (5). Non-invasive diagnostics in some patients, such as scalp electroencephalography (EEG) and MRI are inconclusive or too vague to identify the SOZ. In these cases, intracranial EEG (iEEG) can help to define those brain areas generating seizures.

In the last two decades, high frequency oscillations (HFO; ripples = 80–200, fast ripples 200–500 Hz) became promising biomarkers for epileptic activity. An increased occurrence of HFO in the SOZ could be seen in many clinical studies with patients suffering from medically refractory epilepsy (6–9). Some studies showed that HFO were more specific for the SOZ than interictal epileptic spikes (IES) (10, 11). More importantly, many studies and a meta-analysis showed a correlation of the removal of HFO-generating brain tissue with seizure-free outcome, in some cases even superior to the removal of the SOZ or the Irritative Zone (12–14). However, a multicenter trial and other studies indicated that HFO can only predict outcomes in some but not all patients (13, 15–17), and another study recently showed no benefit of HFO as biomarkers in comparison to IES (18).

When using HFO to delineate the epileptic zone one major problem is the coexistence of epileptic and physiological HFO. Physiological HFO, mainly in the ripple band, can be found in all brain regions (19) and cannot be distinguished from epileptic ripples by their frequency characteristics (20, 21). In neocortical regions, physiological ripples are often seen as task-related or induced by sensory stimulation (22–24). In mesial-temporal regions, physiological ripples play an important part in a fine-tuned mechanism of memory consolidation during non-rapid-eye-movement-sleep (25). During up-states of cortical slow waves (<1 Hz), the thalamus is stimulated to produce cortico-thalamical sleep spindles, transient (0.5–2 sec) oscillatory activity between 12 and 16 Hz, seen in frontal and parietal scalp EEG contacts (26). During these sleep spindles, the thalamic “gate” is closed for sensory input, providing ideal conditions for unimpaired memory transfer via synchronous mesial-temporal ripple activity (27–31).

In patients with epilepsy, the distinction between physiological and epileptic activity is extremely challenging, as epileptic and physiological interictal HFO both occur together in time and space (32). However, the co-occurrence of ripples with scalp EEG spindles enhances the chance to extract physiological activity. Furthermore, ripples co-occurring with sleep spindles showed different amplitude properties than epileptic IES-associated ripples (33, 34). More importantly, coupling of ripples to sleep spindle troughs (i.e. the depolarized down-states of sleep spindle oscillations) could be seen (33, 35). These spindle (-trough) coupled ripples especially form a likely reliable subgroup of physiological ripples.

In the present study, we aim to investigate whether a higher rate of spindle-ripples in mesial-temporal regions correlates with memory function. During the pre-surgical investigation, patients undergo neuropsychological evaluations to identify brain regions that show decreased function due to epileptic activity. Specific memory tests can aid to distinguish between left- and/or right-sided impairment of memory function. In a patient with a left-hemispherical speech-dominance the left temporal lobe including the hippocampus is usually linked to verbal memory function (36), whereas the right hippocampus is linked to non-verbal memory function such as the memory of faces, figures, and shapes (37).

The results of these detailed pre-surgical investigations can be correlated with the amount of HFO visible in the mesial-temporal structures during an iEEG investigation. If we are able to identify a subgroup of HFO (like spindle-ripples) in the hippocampus that is clearly linked to memory function this would allow two crucial advances in HFO research. First, we would improve the differentiation between physiological and epileptic HFO. Second, a subgroup of HFO could be used as a neurophysiological measure of memory function beyond neuropsychological testing.

For this study, we used a unique dataset of patients who underwent iEEG and received neuropsychological testing before and after epilepsy surgery. Regarding ripple rates in mesial-temporal structures of patients with suspected epileptic mesial-temporal lobe involvement, we hypothesize that spindle coupled ripples correlate with pre-surgical memory performance. In consequence, we hypothesize that surgical removal of spindle-ripple-generating tissue results in a deterioration of memory performance. In contrast to spindle-ripples, we hypothesize that the majority of mesial-temporal ripples and IES-associated ripples are epileptic and each correlate with impairment of memory function.

## Methods

### Patient Selection

All patients that underwent chronic intracranial EEG (iEEG) monitoring at Freiburg Epilepsy Center between January 2012 and July 2019 were considered for inclusion. Intracranial EEG was only performed for clinical reasons. Electrode locations were solely selected according to clinical needs and designed in an interdisciplinary surgical conference according to seizure semiology, scalp- EEG, neuroimaging, and neuropsychological evaluation. EEG evaluation and the SOZ determination were performed by experienced neurophysiologists independent of this study. All patients gave written informed consent to the EEG analysis for scientific purposes and the Ethics Committee of the Freiburg University Medical Center agreed to the study.

The inclusion criteria for this study were as follows:

- Unilateral or bilateral depth electrode implantation in mesial-temporal structures (hippocampus and/or parahippocampal gyrus).
- Simultaneous intracranial and scalp EEG recordings.
- EEG sampling rate of at least 2,000 Hz.
- Epilepsy surgery after the iEEG investigation.
- Successfully completed pre- and post-surgical memory assessment at the Freiburg Epilepsy Center.

### Recording Methods

Intracranial depth electrodes were implanted. Electrodes were made of Platinum/Iridium (Dixi Medical, Besancon, France) with five to 18 contacts and a diameter of 0.8 mm. A digital video system called “Profusion EEG Software” (Compumedics Limited, Abbotsford Victoria, Australia) was used to record iEEG. The sampling rate was 2,000 Hz using a digital low-pass filter with a cutoff frequency of 800 Hz. Scalp EEG electrodes were placed according to the international 10–20-system. Electrooculogram and electromyogram were added on the second day after the intracranial electrode implantation. Sleep staging was performed according to the American Academy of Sleep Medicine guidelines by trained technologists.

### EEG Selection

Sleep spindles and ripples are predominantly prevalent in the N2 stage of slow wave sleep (38, 39) therefore only N2 stage periods were chosen. For each patient 30 min of iEEG with a simultaneous scalp EEG was selected. Only periods with at least 1 h distance to subclinical or clinical seizure were considered.

EEG data were converted into binary format and high-pass-filtered using the “ASA” software” (ANT Neuro, Enschede, Netherlands) via 2nd Butterworth filter with a cut-off-frequency of 0.5 Hz. After that, EEG files were transformed into the edf-format. The first 5 min of every EEG were screened by a neurophysiologist and contacts with continuous artifacts in intracranial or scalp EEG were rejected from further analysis.

### Automatic Detection

HFO and IES were automatically detected in all hippocampal and parahippocampal (afterwards stated as mesial-temporal) iEEG channels. Frontal and parietal sleep spindles were detected on the simultaneous scalp EEG. For both analyses, a previously published detector was used (40). This detector is based on the multivariate classification of iEEG epochs using kernelized support-vector-machines.

The ripples used to train the detectors were visually marked by experts. Ripples are oscillations in the iEEG that clearly stand out in amplitude from the basic iEEG activity and consist of at least four consecutive oscillations. IES are fast-transients common to epileptic patients, that are generated by a hyper-excitability of neural networks and have durations shorter than 250 ms. A distinct set of features was calculated for ripples and IES. These features served to characterize iEEG segments/epochs with a duration of 25 ms and 50% overlap with the adjacent epochs, thus providing a 12.5 ms time resolution. The iEEG epochs represented the inputs fed to the support vector machine (SVM classifiers). The classified epochs then formed the ripple and IES detections based on the following rules:

- All epochs generating an output higher or equal to the ripple and IES specific threshold were classified as positive epochs.
- Ripple and IES detections were formed by those immediately adjacent and positive epochs.
- Ripple and IES detections having durations longer than 250 ms were eliminated.
- Detections from the same ripple and IES detections and less than 25 ms apart from each other were joined, if the duration of the joined detection exceeded 250 ms, the lowest start time was kept and a duration of 250 ms was defined.

IES-Ripple consisted of, respectively, ripple detections that originated from the same channel as IES detection and had at least a 75% temporal coincidence with the same IES detection.

The detected sleep spindles were scalp EEG events with an oscillatory waveform, a duration between 0.5 and 3.0 s, and an oscillating frequency between 11 and 16 Hz. The detection was firstly based on the calculation of a set of features that characterized the EEG trace. Each feature was calculated for scalp EEG segments/epochs with a duration of 100 ms. These characterized EEG epochs were the events fed to and classified by the SVM. Once the classification of the scalp EEG segments was done, the sleep-spindle detections were then formed based on the following rules:

- All epochs generating an output higher or equal to the SVM threshold were classified as positive epochs.
- Spindle detections were formed by all of the immediately adjacent positive epochs.
- Spindle detections with a duration shorter than 500 ms were eliminated.
- Spindle detections having a duration longer than 3.0 s were eliminated.
- Spindle detections less than 500 ms apart from each other were joined, if the duration of the joined detection exceeded 3.0s, the lowest start time was kept and a duration of 3.0 s was defined.

The features used for the multivariate classification described the amplitude, waveform, and frequency characteristics [see also (34, 41)] and were also based on the raw, filtered, and wavelet-transformed signals. The description of the feature calculation and selection are described in the corresponding publications, as well as the procedures followed for the training, validation, and testing of the detectors. A custom MATLAB 2018b script was used to determine whether ripples were co-occurring with sleep spindles and IES. Previously published methods from our group were used to determine whether ripples were coupled to spindle troughs (33, 42).

**Figure 1:**
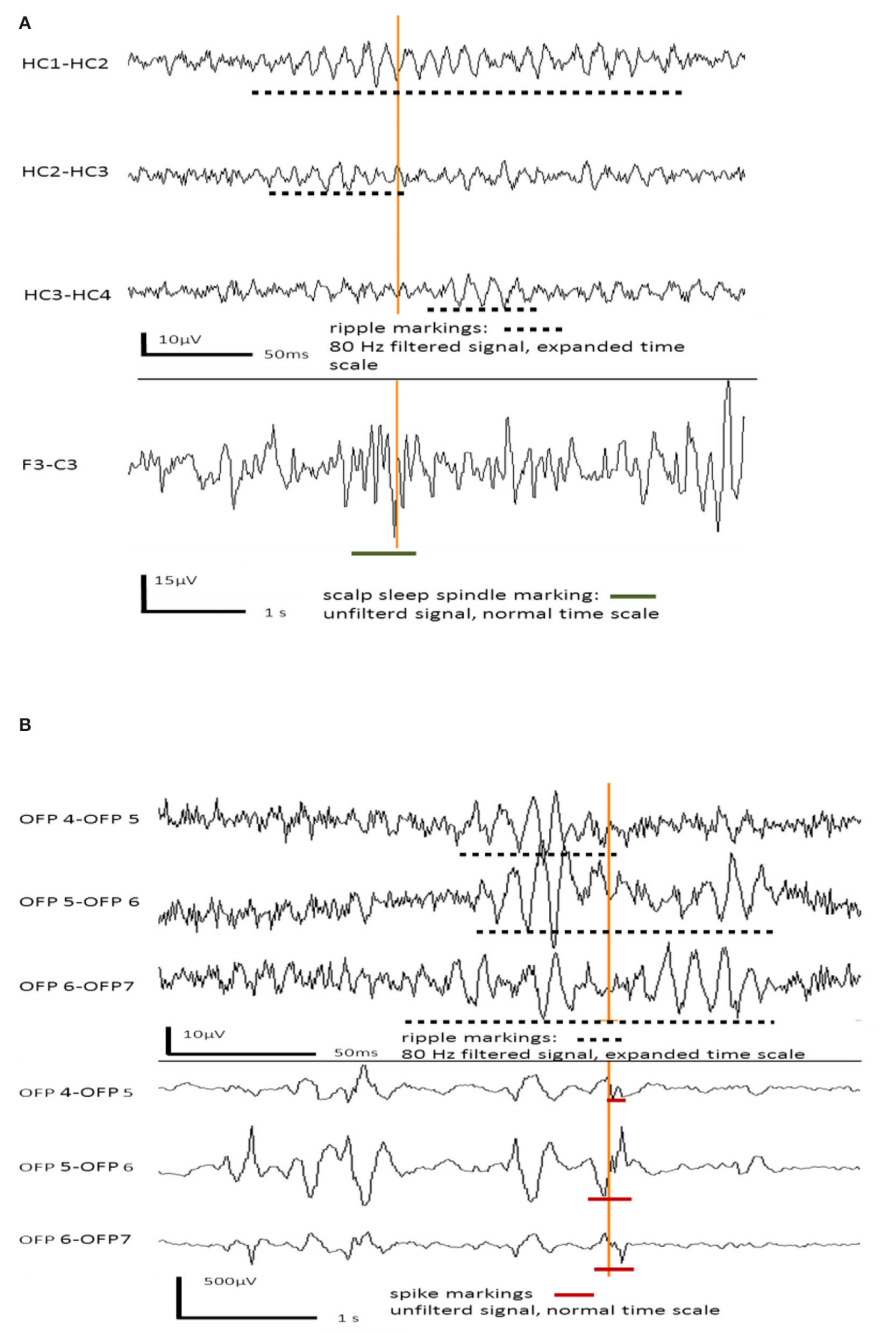
**(A)** Example for hippocampal ripples in intracranial EEG channels co-occurring with a frontal sleep spindle in scalp EEG. **(B)** Example for hippocampal ripples co-occurring with interictal epileptic spikes in the same intracranial EEG channels.

Figure 1A shows an example of an EEG sleep spindle with co-occurring iEEG ripples. Figure 1B shows examples iEEG IES with iEEG ripples.

### Neuropsychological Evaluation

Verbal memory performance was evaluated using the Verbaler Lern- und Merkfaehigkeitstest (VLMT) (43), a modified German version of the Rey Auditory Verbal Learning Test – RAVLT (44). The VLMT tests serial learning and immediate recall of 15 words in five consecutive learning trials, free recall after distraction as well as delayed free recall and recognition of the target words after a 30-min delay. Analysis was based on delayed free recall performance: The number of freely recalled words (trial 7) as well as relative delayed recall performance as compared to the last learning trial (trial 5 minus 7). These parameters have shown to be sensitive to left temporal lobe dysfunction, especially left mesial-temporal pathology and left-sided temporal lobe surgery (45–47).

In order to assess the function of the non-dominant temporal lobe, a figural learning and memory test was used [DCS = Diagnosticum fuer Cerebralschaedigung revised, (48)]. The DCS tests learning and immediate recall of nine abstract designs consisting of five lines in five consecutive trials. Patients are asked to reproduce the designs from memory using five small sticks. Figural learning performance (sum of trials 1 to 5) and figural memory capacity (last learning trial) have been demonstrated to assess functions of the right temporal lobe, including hippocampal functions (49).

Verbal and non-verbal memory performance was assessed pre-surgically as well as approximately 1 year after surgery. Parallel test versions were used in the examination after surgery.

Z-scores were computed based on the VLMT- and DCS raw values and age-matched normative data retrieved from the test manuals. In order to examine post-surgical memory outcome, z-score differences between pre- and post-surgical memory performance were computed. A decrease in z-scores of more than one (standard deviation) was defined as deterioration.

### Statistical Analyses

Three different ripple groups were defined, according to their association with IES and sleep spindles: (1). Overall ripples numbers (= All ripples), (2). ripples co-occurring with IES in the same iEEG channels (= IES ripples) and (3). spindle coupled ripples, i.e. ripples coincident with scalp spindles AND coupled to spindle troughs (=Spindle ripples). In all analyses, these three ripple types were observed separately.

In each patient, the correspondent memory-test results (z-score) were assigned to the respective mean mesial-temporal ripple rate, i.e. verbal memory performance to the left (para)hippocampal ripple rates and non-verbal memory performance to the right (para)hippocampal ripple rates (provided a left-sided speech dominance). Analyses were performed for verbal and non-verbal memory separately. Memory performance with a pre-surgical z-score < −1,0 was evaluated to be “impaired”, and z-scores of −1 and larger as “healthy”.

Three analyses were performed:

1. Mann–Whitney-U-Tests (two-sided) comparing ripple rates between patients with healthy and impaired memory.
2. Spearman correlations (one-sided) between the different ripple rates and the respective pre-surgical verbal or non-verbal memory performance.
3. Spearman correlation analysis of non-SOZ *vs*. SOZ ripples and verbal memory performance.
4. “Delta analysis” using Spearman correlations between the different ripple rates and the difference (“delta”) of post- and pre-surgical memory results (post- minus pre-surgical z-scores).
  - For all patients with left-sided temporal surgery.
  - For patients in whom a surgical intervention had led to a removal of a left-sided (“verbal memory”) hippocampus.

As the attribution of non-verbal memory is questioned, we concentrated on the well-established attribution of verbal memory performance to ripples in iEEG channels in the speech-dominant hippocampus in the “Delta analysis” (for further details, see Discussion).

The significance level of all analyses was α = 0.05.

## Results

### Patients and Clinical Data

A total of 25 patients could be included. Two patients had to be excluded because of a low EEG sample rate and four patients because of artifact-impaired scalp EEG, which prevented spindle analysis. Table 1 offers an overview of the clinical data and implantation sites of the included patients. Two patients had seizures starting bilaterally in the mesial-temporal regions, 17 patients had seizures starting in one mesial-temporal region and six patients had seizure onset in the temporal lobe outside of mesial-temporal regions. Eleven patients received right-sided and six patients received left-sided amygdalohippocampectomy. Eight patients received temporal lobe resections without removing the mesial-temporal structures (four patients: left side, four patients: right side).

**Table 1:** Clinical data.

**Patient**	**Age**	**Gender**	**Seizure type**	**MRI**	**Surgery**	**SOZ**	**Implantation**
1	12	m	FAS, FIAS	tuberous sclerosis	R TL res & SAH	R mTL	R-HC, R-A, R-PHC, R-P, R-F
2	54	m	FIAS, FBTCS	L T-pole MEC, parenchymal lesions R F-bas & L-T-pole	L T-pole res	L mTL	L-HC, L-A, L-PHC
3	33	f	FAS, FIAS, FBTCS	no lesion	R tailored TL res	R TL w/o mTL	R-HC, R-A, R-PHC
4	47	m	FAS, FIAS	BL periventricular heterotopia, L HS	L SAH	L mTL	L-HC, L-A, L-PHC, L-O
5	49	m	FAS, FIAS	no lesion	R SAH	R mTL	L-HC, L-A, L-PHC, R-HC, R-A, R-PHC, R-F
6	53	m	FAS, FIAS, FBTCS	suspected FCD in R T-pole	R T-pole res & SAH	R mTL	R-HC, R-A, R-PHC, R-F
7	27	m	FAS, FBTCS	no lesion	R STG res	R STG	R-HC, R-A, R-PHC, R-F
8	33	m	FAS, FIAS, FBTCS	L mTL FCD	L post HC res & lesionectomy	L mTL	L-HC, L-A, L-PHC, L-F
9	52	f	FAS, FIAS, FBTCS	no lesion	L HC res & lesionectomy	L mTL	L-HC, L-A, L-PHC
10	55	f	FIAS, FBTCS	no lesion	L TL res & SAH	L mTL	L-HC, L-A, L-PHC, L-F, L-O
11	60	f	FAS, FIAS, FBTCS	BL T-pole MEC, L T-pole P lesion	L T-pole res	L T-pole	L-HC, L-A, L-PHC, R-PHC, R-A
12	28	f	FAS, FIAS	no lesion	L SAH	L mTL	L-HC, L-A, L-PHC
13	23	f	FAS, FIAS, FBTCS	R HS	R T-pole res & SAH	R & L mTL, T-pole	L-HC, L-A, L-PHC, R-HC, R-A, R-PHC
14	23	f	FAS, FIAS, FBTCS	no lesion	L SAH	L mTL	L-HC, L-A, L-PHC
15	37	f	FAS, FIAS, FBTCS	R T-pole MEC	R T-pole res	R T-pole	R-HC, R-A, R-PHC, R-T-pole
16	34	m	FAS, FIAS, FBTCS	L T-pole MEC	L T-pole res	L T-pole	L-HC, L-A, L-PHC
17	12	m	FAS, FIAS	suspected FCD in R T-pole	R TL res & SAH	R mTL	R-HC, R-A, R-PHC, R-F
18	21	f	FAS, FIAS, FBTCS	PCA WHO°I	R TL res & SAH	R mTL	R-HC, R-A, R-PHC, R-F
19	34	f	FAS, FIAS, FBTCS	ganglioglioma WHO°I	L FL/TL res & SAH	L FL, TL, mTL	L-HC, L-A, L-PHC, L-F, R-HC, R-A, R-PHC
20	48	m	FAS, FIAS, FBTCS	R F DVA	R O T res	R T O res	L-HC, L-A, L-PHC, R-HC, R-A, R-PHC, R-T, R-O
21	31	f	FAS, FIAS	BL HS	R TL res & SAH	R mTL	R-HC, R-A, R-PHC
22	40	f	FAS, FIAS, FBTCS	R HS	R TL res & SAH	R mTL	R-HC, R-A, R-PHC
23	44	f	FAS, FIAS, FBTCS	BL T P MEC	L T-pole res	L T-pole	L-HC, L-A, L-PHC, R-HC, R-A, R-PHC
24	45	m	FIAS, FBTCS	no temporal lesion	R TL res & SAH	R & L mTL	L-HC, L-A, L-PHC, R-HC, R-A, R-PHC
25	22	m	FAS	R HS	R SAH	R mTL	R-HC, R-A, R-PHC, R-F, R-P

***Abbreviations:** A, Amygdala; AC, arachnoid cyst; SAH, selective amygdalohippocampectomy; bas, basal; BL, bilateral; DVA, developmental venous anomaly; FL, frontal lobe; FAS, focal aware seizure; FBTCS, focal to bilateral tonic-clonic seizure; FCD, focal cortical dysplasia; FIAS, focal impaired awareness seizure; HC, hippocampal, HS, hippocampal sclerosis; L, left; MEC, meningoencephalocele; mTL, mesial temporal lobe; O, occipital; P, parietal; PCA, pylocytic astrocytoma; PHC, parahippocampal; post, posterior; R, right; res, resection; STG, superior temporal gyrus; T , temporal; TL, temporal lobe*.

### Memory Performance

In pre-surgical memory testing nine patients had significant non-verbal memory impairment (Z-score < −1,0) with intact verbal memory, one patient had verbal memory impairment with intact non-verbal memory and four patients had both verbal and non-verbal memory impairment.

Of the 11 patients with right-sided amygdalohippocampectomy, seven patients had similar memory performance outcomes and one patient had a post-surgical deterioration of non-verbal memory function. Two patients benefitted from surgery with amelioration of non-verbal memory function.

Of the six patients with left-sided amygdalohippocampectomy, one patient had a similar memory performance outcome and three patients had deterioration of verbal memory function (two patients in verbal and one patient in verbal and non-verbal memory). In two patients with left-sided amygdalohippocampectomy a significant amelioration of non-verbal memory was observed, whilst verbal memory remained impaired in these patients. Table 2 gives an overview of the memory performance results of the cohort.

**Table 2:** Memory performance results.

**Patient**	**Speech**	**IQ**	**Pre VM**	**Pre NVM**	**Post VM**	**Post NVM**	**Surgery**
1	L	N.A.	healthy	impaired	healthy	impaired	R TL incl MTL
2	L	109	healthy	impaired	healthy	healthy	L T-pole (MTL pres)
3	L	104	healthy	healthy	healthy	healthy	tailored TL res
4	L	100	impaired	impaired	impaired	healthy	L sAHE
5	L	107	healthy	healthy	healthy	healthy	R sAHE
6	L	N.A.	healthy	healthy	healthy	impaired	R T-pole res & AHE
7	L	124	healthy	healthy	healthy	healthy	R STG res
8	L	94	healthy	healthy	impaired	healthy	L post HC res & lesionectomy
9	L	118	healthy	impaired	impaired	impaired	L HC res & lesionectomy
10	L	111	impaired	impaired	impaired	impaired	L TL res & sAHE
11	L	100	healthy	impaired	impaired	N.A.	L T-pole res
12	L	101	healthy	healthy	impaired	impaired	L sAHE
13	L	93	healthy	impaired	healthy	impaired	R T-pole res & AHE
14	L	92	impaired	impaired	impaired	healthy	L sAHE
15	L	N.A.	healthy	healthy	healthy	healthy	R T-pole res
16	L	95	healthy	N.A.	healthy	healthy	L T-pole res
17	L	N.A.	healthy	healthy	healthy	healthy	R TL res & AHE
18	L	121	healthy	healthy	healthy	healthy	R TL res & AHE
19	L	81	impaired	impaired	impaired	impaired	R TL res & AHE
20	L	143	healthy	healthy	healthy	healthy	R O T res
21	L	104	healthy	impaired	healthy	impaired	R TL res & AHE
22	L	N.A.	healthy	impaired	healthy	healthy	R TL res & AHE
23	L	93	healthy	impaired	healthy	impaired	L T-pole res
24	L	N.A.	healthy	impaired	healthy	N.A.	R TL res & AHE
25	L	97	impaired	healthy	healthy	healthy	R sAHE

***Abbreviations:** L, left; rem mTL, remaining mesiotemporal structures; (m)TL, (mesial) temporal lobe; N.A., not available; NVM, Nonverbal Memory; Pre, pre-surgical; Post,post-surgical; VM, Verbal Memory; R,right*.

### Descriptive Ripple Statistics

Ripples, IES-associated ripples, and spindle-coupled ripples were found in all patients. In total, 231 mesial-temporal channels were analyzed, including 95 SOZ channels and 136 channels outside the SOZ (NSOZ). The following ripple rates are given as mean ± standard deviation with the interquartile ranges in brackets. Considering all channels, rates of 14.1 ± 9.5/min (15,0/min) overall ripples, 6.2 ± 6.1/min (9.0/min) IES ripples and 0.9 ± 1.0/min (0.7/min) spindle coupled ripples and 7.9 ± 5.0/min (7.1/min) isolated ripples (without any co-occurrence with sleep-spindles or IES showed) were detected.

In SOZ channels rates of 18.5 ± 1.5/min (9.1/min) overall ripples, 9.1 ± 6.8/min (10.8/min) IES ripples and 1.2 ± 0.7/min (1.2/min) spindle coupled ripples were detected. In NSOZ channels rates of 11.0 ± 8.0/min (11.9/min) overall ripples, 4.3 ± 5.0/min (6.1/min) IES ripples and 0.8 ± 0.7/min (0.7/min) spindle coupled ripples were detected. The rates of all three ripple types were significantly higher in SOZ channels than in NSOZ channels (*p* < 0.001 for each ripple type).

### Comparison Pre-surgical Memory Impairment and Ripple Rates

To examine a connection between pre-surgical memory impairment and mesial-temporal ripple rates, patients were divided into two groups (healthy vs. impaired). Impaired memory was defined by a memory performance z-score under −1,0. Mann–Whitney-U-Tests were computed separately for verbal and non-verbal memory performances, attributing left mesial-temporal ripple rates to verbal memory and right mesial-temporal ripple rates to non-verbal memory.

Regarding verbal memory and left-sided mesial-temporal ripple rates, significantly higher overall ripple rates were seen in patients with impaired verbal memory than in patients with healthy memory (*p* = 0.039). Regarding IES ripples, higher rates were also seen in patients with impaired verbal memory, but the difference was not significant (*p* = 0.078). Regarding spindle ripples, no differences between the two groups were seen (see Figure 3A).

Regarding non-verbal memory and right-sided mesial-temporal ripple rates, no differences between the two groups were seen in all three ripple types (All Ripples: *p* = 0.606; IES Ripples: *p* = 0.541; Spindle Ripples: *p* = 0.423) (see Figure 3B).

### Correlation Analysis of Pre-surgical Memory Performance and Ripple Rates

This analysis was performed to investigate the assumed correlation of ripples, especially spindle-ripples, and pre-surgical memory performance.

Spearman correlations of left mesial-temporal ripple rates and pre-surgical verbal memory results showed a highly significant negative correlation of the overall ripple rate and pre-surgical verbal memory performance (z-scores) was seen (r_s_= −0.475; *p* = 0.006) (see Figure 4A). For IES ripples, a negative correlation with pre-surgical memory was seen as well (r_s_= −0.458; *p* = 0.007) (see Figure 4B), whereas there was no significant correlation between spindle ripples and verbal memory performance (r_s_= −0.194; *p* = 0.149) (see Figure 4C).

Spearman correlations of right mesial-temporal ripple rates and pre-surgical non-verbal memory results (Z-scores) showed no correlation regarding overall ripples (r_s_ = 0.160; *p* = 0.540) and IES ripples (r_s_ = 0.119; *p* = 0.377). No significant correlation for spindle ripples was seen either (r_s_= 0.324; *p* = 0.204).

### Correlation Analysis of Non-SOZ vs. SOZ Ripples and Verbal Memory Performance

**Figure 2:**
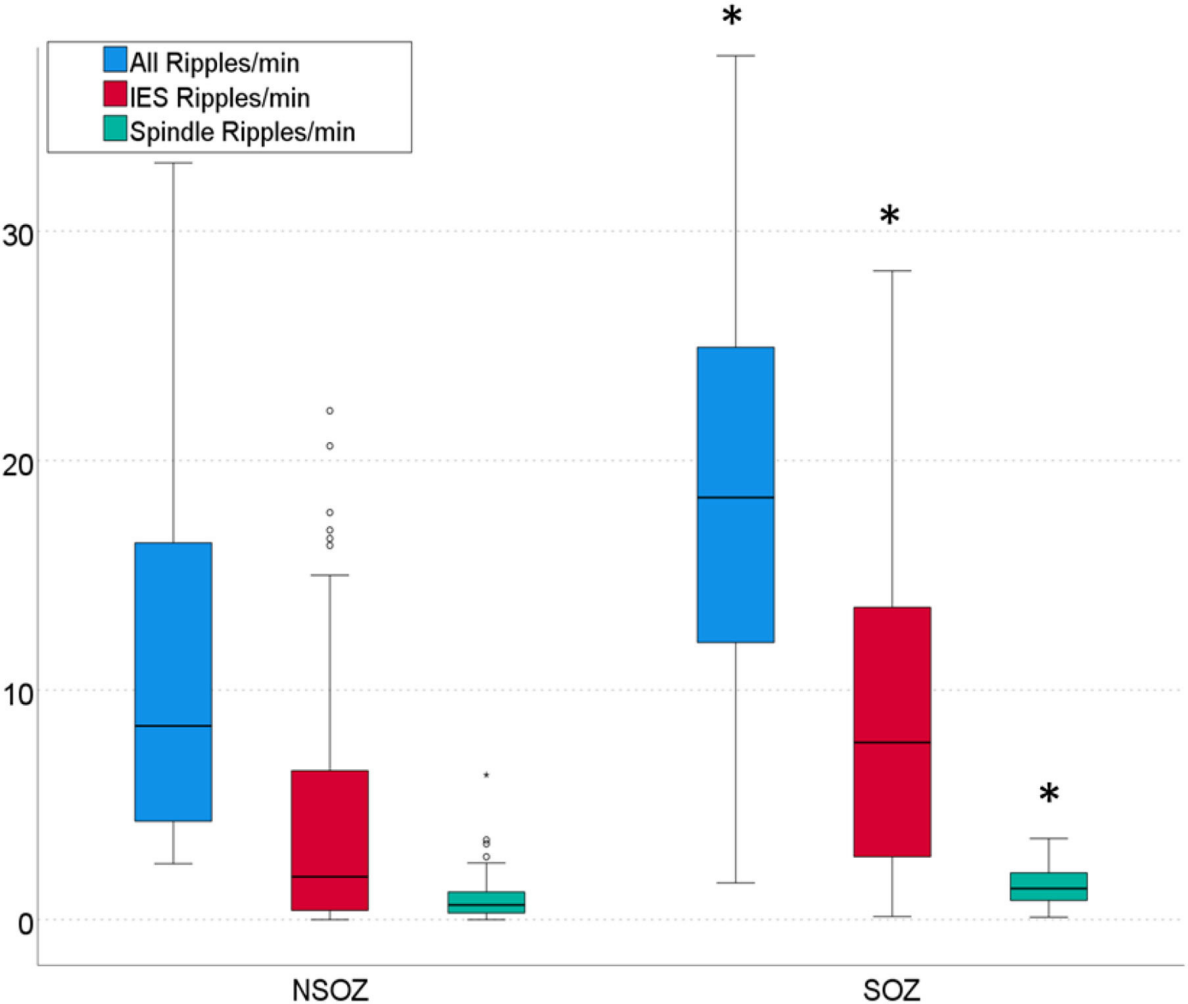
Rates of three ripple types per min in channels outside the seizure onset zone (NSOZ) compared to ripples in channels inside the seizure onset zone (SOZ). Significantly higher overall ripple, IES ripple, and Spindle ripple rates were found in SOZ channels (^*^p < 0.001, respectively).

Focussing on the verbal side hippocampal SOZ channels, Spearman correlations were performed regarding a possible connection of different ripple events and z-values of verbal memory performance. In this analysis, no significant correlation was seen for spindle ripples as expected (rs = −0.144, *p* = 0.337. Regarding overall ripples and IES ripples medium strength negative correlations of ripple rates and memory performance were seen that were not (but tendentially) significant (overall ripples: rs = −0.366; *p* = 0.086; IES ripples: rs = −0.370; *p* = 0.080) (see Figure 2).

**Figure 3:**
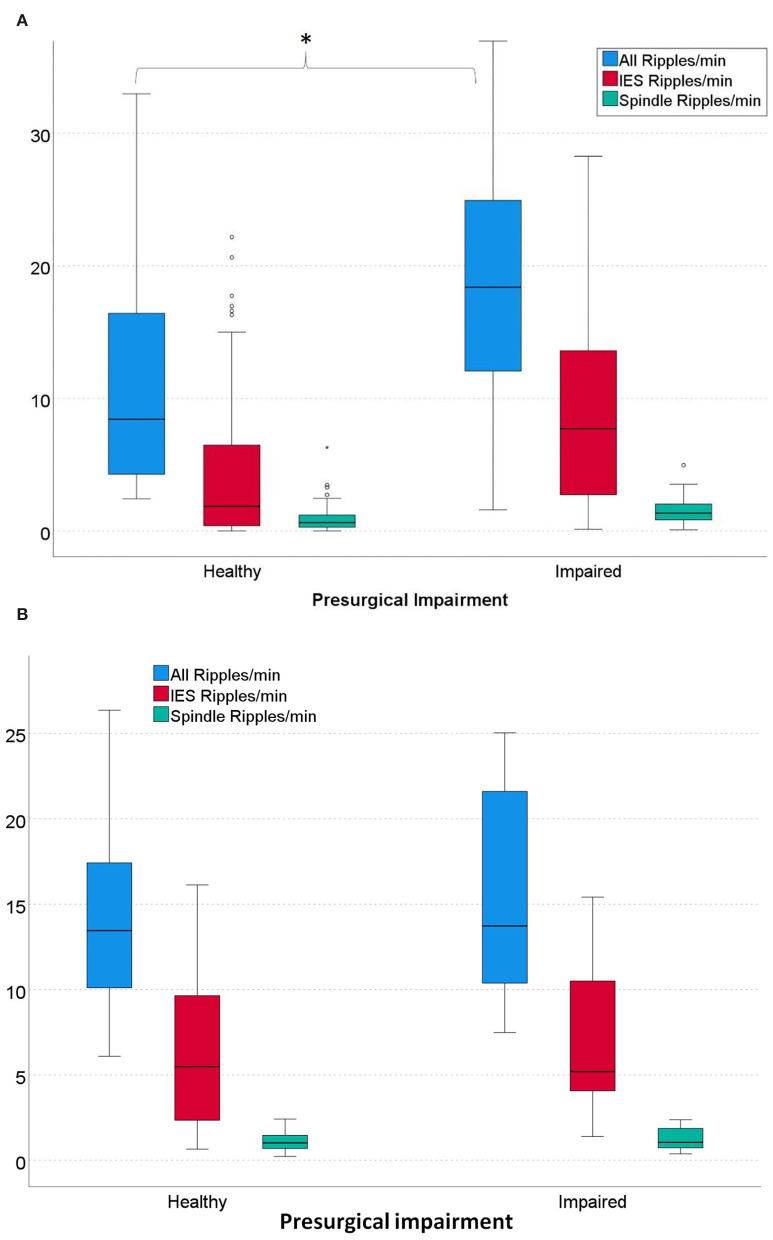
**(A)** Rates of left-sided mesial-temporal ripples in patients with healthy *vs*. impaired pre-surgical verbal memory. Patients with impaired verbal memory had significantly higher overall ripple rates than patients with healthy memory. (All Ripples: *p = 0.039; IES Ripples: *p* = 0.078; Spindle ripples: *p* = 0.379). α = 0.05. **(B)** Rates of all right-sided mesial-temporal overall Ripples and IES Ripples in patients with healthy *vs*. impaired pre-surgical non-verbal memory. No significant differences were seen for all three ripple types (All Ripples: *p* = 0.606; IES Ripples: *p* = 0.541; Spindle Ripples: *p* = 0.423); α = 0.05.

**Figure 4:**
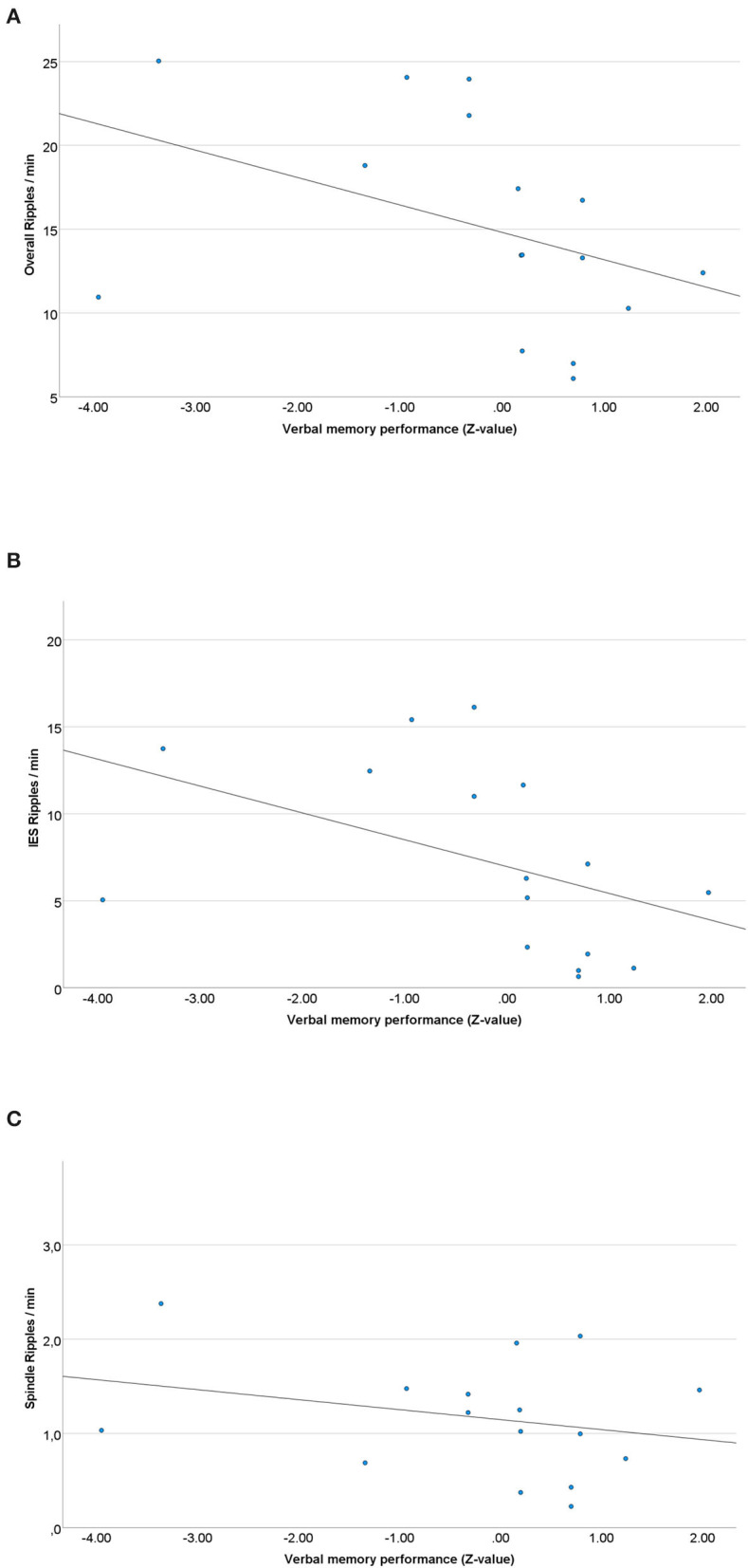
**(A)** Scatterplot of the overall left mesial-temporal ripple rate and pre-surgical verbal memory results. A significant negative correlation between ripple rates and memory performance was seen (r_s_ = −0.475; *p* = 0.006). **(B)** Scatterplot of the left mesial-temporal IES ripple rate and pre-surgical verbal memory results. A significant negative Spearman correlation was seen (r_s_ = −0.458; *p* = 0.007). **(C)** Scatterplot of the left sided spindle ripple rate and pre-surgical verbal memory results. No significant Spearman correlation was seen (r_s_ = −0.194; *p* = 0.149).

### Delta Analysis (Correlation Analysis of Post- to Pre-surgical Verbal Memory Difference and Ripple Rates)

In the following analysis, we aimed to examine the influence of surgical removal of ripple generating tissue on post-surgical verbal memory performance. Therefore, Spearman correlations between the difference in post- to pre-surgical verbal memory performance and left-sided ripple rates were computed. The 16 patients with left-sided temporal or mesial-temporal surgery were included in this analysis. No significant correlation was seen regarding all three ripple rates (all ripples: r_s_= 0.092; *p* = 0.310; IES ripples: r_s_= 0.059; *p* = 0.376; spindle ripples: r_s_ = 0.042; *p* = 0.411).

In a sub-analysis only the six patients with left-sided amygdalohippocampectomy were considered. Again, no correlation between the difference of post- and pre-surgical memory performance [Δ = post-VLMT - pre-VLMT(Z-scores)] and the rates of all three ripple types was seen (all ripples: r_s_= 0.127; *p* = 0.293; IES ripples: r_s_ = 0.018; *p* = 0.469; spindle ripples: r_s_= 0.220; *p* = 0.174).

## Discussion

The main hypothesis of this study was that higher rates of mesial-temporal spindle-ripples correlate with good memory performance in epilepsy patients. In addition, we hypothesized that surgical removal of spindle-ripple-generating brain tissue would correlate with a decline in memory performance. In contrast, higher rates of ripples associated with IES were hypothesized to correlate with poor memory performance and the surgical removal of IES ripples would lead to an enhancement of memory performance.

A significant correlation between spindle ripples and pre-surgical verbal or non-verbal memory performance could not be found in this study. Removing tissue that generated high rates of spindle-ripples showed no correlation to decline of memory function. As hypothesized, high ripple rates of overall ripples and IES ripples were associated with poor memory performance. This indicates that the majority of ripples identified in our patients are linked to epileptic activity. Most likely the percentage of physiological spindle-ripples that contribute to memory function is too small compared to the overall ripple population and therefore hard to identify in mesial-temporal structures of patients with severe epilepsy.

### Methodological Considerations

Ripple, IES, and sleep spindle detection were performed automatically by a published detector of our group (40). In addition, the first 5 min of each EEG segment were visually checked for artifacts. In contrast to time-consuming visual detection, this semi-automatic approach would be applicable for potential clinical use. We chose relatively short segments of N2 periods of slow-wave sleep, as an extension of record time would not lead to a higher share of spindle ripple rates and would have extended the difficulty of finding interictal periods outside of seizures and subclinical seizures.

In other studies observing physiological HFO activities, simultaneously performed neuropsychological tasks were used to induce or increase physiological HFO activity (22, 24, 50, 51). In this study, data was collected retrospectively and no specific memory tasks were conducted prior to the iEEG recording nights due to two reasons: On the one hand, it can be assumed that patients learn new memory content every day and spindle-ripple activity associated with memory consolidation can be observed without extra memory tasks. On the other hand, this study aimed to estimate the impact of physiological oscillations on the evaluation of a standard iEEG during the recording period, as this is the information needed by clinicians when evaluating ripples as markers of epileptogenic brain regions.

One challenge of the present study is to clearly separate areas that have a high function from those that generate seizures. Healthy and epileptic regions in patients with refractory epilepsy often overlap and seizures arise from hippocampi that can still have good remaining memory function. Usually, only patients with contradictory results in prior diagnostics (scalp EEG, MRI, neuropsychological tests) receive iEEG diagnostics. This leads to a pre-selection of patients with highly complicated epilepsies and often bilateral mesial-temporal involvement.

Especially in temporal lobe epilepsy, clinicians and researchers face an extensively complicated network disease (52–54). Bilateral independent seizures are frequent (55). Regularly, patients with unilateral surgical removal of the diagnosed epileptogenic zone do not experience seizure reduction after unilateral mesial-temporal lobe (MTL) resection (55–57). This suggests that epileptic regions could remain off the grid, e.g. one potential epileptic hippocampus could not be considered as part of the SOZ in the short period of iEEG registration. Furthermore, there are no secure healthy control regions, concepts of SOZ, and areas outside the SOZ used in this study are susceptible to errors and uncertainties. In our study, this is directly reflected in the results as it is challenging to separate ripples reflecting epileptic activity from those representing memory.

Regarding our delta analysis (analyzing pre- and post-surgical memory), we intended to obtain a preferably clear attribution of ripple generating tissue to a certain brain region. It has been discussed if the type of material used during a memory task as well as the underlying processing influence the functional asymmetry in the mesial-temporal lobe and therefore challenges the left-verbal/right-non-verbal dichotomy (58). Especially the attribution of the non-speech-dominant hippocampus to non-verbal memory is questioned (59). Therefore, we concentrated on the well-established attribution of verbal memory performance to ripples in iEEG channels in the speech-dominant hippocampus. In the cohort of this study all patients had left-sided language dominance, therefore only verbal memory test results and left hippocampal and parahippocampal channels were considered for the delta analysis.

We focused on ripple range HFO for this study, as we were specifically aiming at the co-occurrence between ripples and sleep spindles. No association of fast ripples with sleep-spindles, slow-waves, or another hint of physiological function in the mesial temporal regions has been reported in the literature so far. Most physiological fast ripples are described in paracentral and occipital regions. Also, this phenomenon has nearly exclusively been observed in the context of prior tasks or induction (22, 24).

This study concentrated on memory parameters that are associated with hippocampal memory functions. However, long-term memory consolidation is not only based on mesial temporal lobe function but also on an extensively and bilaterally distributed process (60). Long-term memory storage depends on a functioning precedent of short-term memory storage, e.g. the short-term maintenance of verbal memory occurs in left neocortical networks (left pre-central, supra-marginal, and inferior frontal gyri) (61). An impaired short-term memory process is a possible cause for impairment of long-term memory consolidation. However, the chronological sequence of the overall memory storage process was not investigated. As clinically guided electrode implantation leads to restricted registration areas, we concentrated on spontaneous ripple activity in mesial temporal areas and its attributed memory functions. Impaired short-term memory therefore could be one reason, why a correlation between memory performance and spindle ripple rates was not found in this study.

In addition, the (long-term) influence of anti-epileptic drugs on memory capability is unclear. Most of the patients in iEEG studies have taken multiple anti-epileptic-drugs medications. This could be another circumstance that had a perturbing influence on this study.

### Mesial-Temporal Physiological and Epileptic Ripples

Few studies have examined the interaction of memory performance and mesial-temporal ripples in general or spindle-associated ripples in particular. Axmacher and co-workers found an increase in overall ripple band HFO during cognitive tasks and were the first to find a correlation between ripple rates and memory performance in humans (50). A recent study found a correlation between the amount of spindle coincident ripples after a spatial navigation task in two subsequent iEEG recording nights (51). Thus, in addition to the knowledge of spindle-associated ripple activity (33, 35, 62), there seems to be strong evidence that physiological ripples can be observed in mesial-temporal structures of patients with epilepsy. However, a correlation between spindle-trough-coupled ripples and memory function could not be seen in the current study. Nevertheless, it seems likely, that the detected spindle ripples mirror physiological HFO activity, as the majority of spindle coincident ripples have shown to have different amplitude features and are coupled to spindle troughs (33, 42).

However, it has to be considered that sleep spindles and sharp wave ripples might be pathologically compromised in patients with epilepsy. Transformation of physiologically sharp-wave-ripple complexes or possible pathological hippocampal sleep-spindle activity leading to interictal epileptiform discharges (IED) has been reported in some patients (63, 64). Frauscher and co-workers have particularly observed the relationship between mesial-temporal epilepsy and sleep-spindle expression, showing that hippocampal spiking correlates with lower rates of hippocampal sleep-spindles. The authors suggested that epileptic discharges might be transformations of physiological spindle activity in patients with mesial-temporal lobe epilepsy (65). In the current study, one patient was excluded because of pathologically changed sleep-spindles with sharp transients. In all other patients, sleep-spindles had typical shapes. When pathologically “distorted” spindles are observed, spindle-ripple analysis seems not to be reasonable. However, epileptically compromised sleep-spindle activity seems to be rare. The findings of Frauscher and co-workers might suggest, that in patients with epilepsy the proportion of spindle-ripples might not only be much lower compared to epileptic ripple activity but might also be suppressed by it.

We found a clear negative correlation between pre-surgical memory performance and overall mesial-temporal ripple rates (i.e. irrespective of any coincidence with spindles or IES). A former study showed similar results: Jacobs and co-workers found no correlation between overall HFO or ripple activity and memory performance. On the contrary, a high number of HFO or ripples correlated with poor memory performance outside the SOZ, whereas patients with a good memory performance showed less frequent HFO in channels outside the SOZ (32). It seems that the majority of mesial-temporal ripples in epilepsy patients reflect the pathological activity. In the current study, the share of spindle-ripples was marginal compared to overall ripple rates and much lower than IES-associated ripples. As we could not find any correlation between spindle-ripples and memory, it seems likely, that epileptic ripple activity impairs physiological activity immensely as they co-occur close-by both in time and place, separating these activities.

Removing tissue that showed high rates of spindle ripples prior to resection showed no correlation to a decline of post-surgical memory function in our two groups of patients with left-sided temporal surgery or mesial-temporal surgery. This goes in hand with the findings of a recent study which showed that the value of spindle-ripples alone to identify physiological ripples on pre-surgical diagnostics is limited (66).

## Conclusion

This study fails to establish a clear link between memory performance and spindle-ripples. This highly suggests that spindle-ripples are only a small portion of physiological ripples contributing to memory performance. Standardized neuropsychological tests likely measure functional results of all ripples and might not be sensitive enough to detect changes in spindle-ripple occurrence alone. Moreover, high numbers of epileptic ripples might prevent us from seeing the effect of ripple numbers on memory, especially in patients with bilateral disease and very active epilepsy. However, an important take home message is that our results suggest that spindle ripples do not necessarily compromise the predictive value of ripples in patients with temporal epilepsy. To come closer to our aim of measuring memory function and epileptic activity with HFO, measures beyond spindle-ripples should be evaluated. A combined analysis that includes all known characteristics of physiological ripples, their coupling to sleep spindles and slow waves (67, 68), and additionally considering hippocampal theta waves and other iEEG features (69, 70) might show a better correlation between these ripples and memory function.

## Data Availability Statement

The original contributions presented in the study are included in the article/Supplementary Material, further inquiries can be directed to the corresponding author/s.

## Ethics Statement

The studies involving human participants were reviewed and approved by Ethics Committee of the Freiburg University Medical Center. Written informed consent to participate in this study was provided by the participants' legal guardian/next of kin.

## Author Contributions

JJ, KW, and JB have contributed to the conception and design of the study. JB, KW, and DL-P have contributed to the acquisition and analysis of data. JB, KW, KK, and JJ have drafted significant portions of the manuscript and figures. AS-B provided all clinical data and EEG data. All authors contributed to the article and approved the submitted version.

## Funding

JB, DL-P, and JJ were supported by grant JA 1725/4-1 of the German Research Foundation.

## Conflict of Interest

The authors declare that the research was conducted in the absence of any commercial or financial relationships that could be construed as a potential conflict of interest.

## Publisher's Note

All claims expressed in this article are solely those of the authors and do not necessarily represent those of their affiliated organizations, or those of the publisher, the editors and the reviewers. Any product that may be evaluated in this article, or claim that may be made by its manufacturer, is not guaranteed or endorsed by the publisher.

## References

[1] Kwan P Brodie MJ. Early identification of refractory epilepsy. New England J Med. (2000) 342:314–9. 10.1056/NEJM200002033420503 10660394

[2] Blümcke I Thom M Wiestler OD. Ammon's horn sclerosis: a maldevelopmental disorder associated with temporal lobe epilepsy. Brain Pathol. (2002) 12:199–211. 10.1111/j.1750-3639.2002.tb00436.x 11958375 PMC8095862

[3] Asadi-Pooya AA Stewart GR Abrams DJ Sharan A. Prevalence and Incidence of Drug-Resistant Mesial Temporal Lobe Epilepsy in the United States. World Neurosurg. (2017) 99:662–6. 10.1016/j.wneu.2016.12.074 28034810

[4] Wiebe S Blume WT Girvin JP Eliasziw M. Effectiveness and efficiency of surgery for temporal lobe epilepsy study group. A randomized, controlled trial of surgery for temporal-lobe epilepsy. N Engl J Med. (2001) 345:311–8. 10.1056/NEJM200108023450501 11484687

[5] Rosenow F Lüders H. Presurgical evaluation of epilepsy. Brain. (2001) 124:1683–700. 10.1093/brain/124.9.1683 11522572

[6] Staba RJ Wilson CL Bragin A Fried I Engel J. Quantitative analysis of high-frequency oscillations (80–500 hz) recorded in human epileptic hippocampus and entorhinal cortex. J Neurophysiol. (2002) 88:1743–52. 10.1152/jn.2002.88.4.1743 12364503

[7] Jirsch JD Urrestarazu E LeVan P Olivier A Dubeau F Gotman J. High-frequency oscillations during human focal seizures. Brain. (2006) 129:1593–608. 10.1093/brain/awl085 16632553

[8] Worrell GA Gardner AB Stead SM Hu S Goerss S Cascino GJ. High-frequency oscillations in human temporal lobe: simultaneous microwire and clinical macroelectrode recordings. Brain. (2008) 131:928–37. 10.1093/brain/awn006 18263625 PMC2760070

[9] Jacobs J LeVan P Châtillon C-É Olivier A Dubeau F Gotman J. High frequency oscillations in intracranial EEGs mark epileptogenicity rather than lesion type. Brain. (2009) 132:1022–37. 10.1093/brain/awn351 19297507 PMC3792079

[10] Jacobs J LeVan P Chander R Hall J Dubeau F Gotman J. Interictal high-frequency oscillations (80–500 Hz) are an indicator of seizure onset areas independent of spikes in the human epileptic brain. Epilepsia. (2008) 49:1893–907. 10.1111/j.1528-1167.2008.01656.x 18479382 PMC3792077

[11] Jacobs J Kobayashi K Gotman J. High-frequency changes during interictal spikes detected by time-frequency analysis. Clin Neurophysiol. (2011) 122:32–42. 10.1016/j.clinph.2010.05.033 20599418 PMC3774652

[12] Wu JY Sankar R Lerner JT Matsumoto JH Vinters HV Mathern GW. Removing interictal fast ripples on electrocorticography linked with seizure freedom in children. Neurology. (2010) 75:1686–94. 10.1212/WNL.0b013e3181fc27d0 20926787 PMC3033604

[13] Jacobs J Zijlmans M Zelmann R Chatillon C-E Hall J Olivier A. High-frequency electroencephalographic oscillations correlate with outcome of epilepsy surgery. Ann Neurol. (2010) 67:209–20. 10.1002/ana.21847 20225281 PMC3769290

[14] Höller Y Kutil R Klaffenböck L Thomschewski A Höller PM Bathke AC. High-frequency oscillations in epilepsy and surgical outcome. A meta-analysis. Front Hum Neurosci. (2015) 9:574. 10.3389/fnhum.2015.00574 26539097 PMC4611152

[15] Jacobs J Wu JY Perucca P Zelmann R Mader M Dubeau F. Removing high-frequency oscillations: a prospective multicenter study on seizure outcome. Neurology. (2018) 91:e1040–52. 10.1212/WNL.0000000000006158 30120133 PMC6140372

[16] Haegelen C Perucca P Châtillon C-E Andrade-Valença L Zelmann R Jacobs J. High-frequency oscillations, extent of surgical resection, and surgical outcome in drug-resistant focal epilepsy. Epilepsia. (2013) 54:848–57. 10.1111/epi.12075 23294353 PMC3712982

[17] Fedele T Burnos S Boran E Krayenbühl N Hilfiker P Grunwald T. Resection of high frequency oscillations predicts seizure outcome in the individual patient. Sci Rep. (2017) 7:13836. 10.1038/s41598-017-13064-1 29062105 PMC5653833

[18] Roehri N Bartolomei F. Are high-frequency oscillations better biomarkers of the epileptogenic zone than spikes? Curr Opin Neurol. (2019) 32:213–9. 10.1097/WCO.0000000000000663 30694920

[19] Frauscher B Ellenrieder N von Zelmann R Rogers C Nguyen DK Kahane P. High-frequency oscillations in the normal human brain. Ann Neurol. (2018) 84:374–85. 10.1002/ana.25304 30051505

[20] Engel J Bragin A Staba R Mody I. High-frequency oscillations: what is normal and what is not? Epilepsia. (2009) 50:598–604. 10.1111/j.1528-1167.2008.01917.x 19055491

[21] Fink CG Gliske S Catoni N Stacey WC. Network mechanisms generating abnormal and normal hippocampal high-frequency oscillations: a computational analysis. eNeuro. (2015) 2. 10.1523/ENEURO.0024-15.2015 26146658 PMC4487885

[22] Curio G. High frequency (600 Hz) bursts of spike-like activities generated in the human cerebral somatosensory system. Electroencephalogr Clin Neurophysiol Suppl. (1999) 49:56–61. 10533086

[23] Boran E Stieglitz L Sarnthein J. Epileptic high-frequency oscillations in intracranial EEG are not confounded by cognitive tasks. Front Hum Neurosci. (2021) 15:613125. 10.3389/fnhum.2021.613125 33746723 PMC7971186

[24] Nagasawa T Juhász C Rothermel R Hoechstetter K Sood S Asano E. Spontaneous and visually driven high-frequency oscillations in the occipital cortex: intracranial recording in epileptic patients. Hum Brain Mapp. (2012) 33:569–83. 10.1002/hbm.21233 21432945 PMC3220781

[25] Diekelmann S Born J. The memory function of sleep. Nat Rev Neurosci. (2010) 11:114–26. 10.1038/nrn2762 20046194

[26] Steriade M. Grouping of brain rhythms in corticothalamic systems. Neuroscience. (2006) 137:1087–106. 10.1016/j.neuroscience.2005.10.029 16343791

[27] Siapas AG Wilson MA. Coordinated interactions between hippocampal ripples and cortical spindles during slow-wave sleep. Neuron. (1998) 21:1123–8. 10.1016/S0896-6273(00)80629-7 9856467

[28] Sirota A Csicsvari J Buhl D Buzsáki G. Communication between neocortex and hippocampus during sleep in rodents. Proc Natl Acad Sci U S A. (2003) 100:2065–9. 10.1073/pnas.0437938100 12576550 PMC149959

[29] Clemens Z Mölle M Eross L Barsi P Halász P Born J. Temporal coupling of parahippocampal ripples, sleep spindles and slow oscillations in humans. Brain. (2007) 130:2868–78. 10.1093/brain/awm146 17615093

[30] Fedele T van't Klooster M Burnos S Zweiphenning W van Klink N Leijten F. Automatic detection of high frequency oscillations during epilepsy surgery predicts seizure outcome. Clin Neurophysiol. (2016) 127:3066–74. 10.1016/j.clinph.2016.06.009 27472542

[31] Ngo H-V Fell J Staresina B. Sleep spindles mediate hippocampal-neocortical coupling during long-duration ripples. eLife. (2020) 9:e57011. 10.7554/eLife.57011 32657268 PMC7363445

[32] Jacobs J Banks S Zelmann R Zijlmans M Jones-Gotman M Gotman J. Spontaneous ripples in the hippocampus correlate with epileptogenicity and not memory function in patients with refractory epilepsy. Epilepsy Behav. (2016) 62:258–66. 10.1016/j.yebeh.2016.05.025 27517349

[33] Bruder JC Dümpelmann M Piza DL Mader M Schulze-Bonhage A Jacobs-Le Van J. Physiological ripples associated with sleep spindles differ in waveform morphology from epileptic ripples. Int J Neural Syst. (2016) 2:1750011. 10.1142/S0129065717500113 28043201

[34] Blanco JA Stead M Krieger A Viventi J Marsh WR Lee KH. Unsupervised classification of high-frequency oscillations in human neocortical epilepsy and control patients. J Neurophysiol. (2010) 104:2900–12. 10.1152/jn.01082.2009 20810694 PMC2997042

[35] Staresina BP Bergmann TO Bonnefond M van der Meij R Jensen O Deuker L. Hierarchical nesting of slow oscillations, spindles and ripples in the human hippocampus during sleep. Nat Neurosci. (2015) 18:1679–86. 10.1038/nn.4119 26389842 PMC4625581

[36] Loring DW Strauss E Hermann BP Barr WB Perrine K Trenerry MR. Differential neuropsychological test sensitivity to left temporal lobe epilepsy. J Int Neuropsychol Soc. (2008) 14:394–400. 10.1017/S1355617708080582 18419838

[37] Majdan A Sziklas V Jones-gotman M. Performance of healthy subjects and patients with resection from the anterior temporal lobe on matched tests of verbal and visuoperceptual learning. J Clin Exper Neuropsychol. (1996) 18:416–30. 10.1080/01688639608408998 8877625

[38] Rechtschaffen A Kales A. A Manual of Standardized Terminology, Techniques and Scoring System of Sleep Stages In Human Subjects. Los Angeles: Brain Information Service/Brain Research Institute, University of California. (1968).

[39] De Gennaro L Ferrara M. Sleep spindles: an overview. Sleep Med Rev. (2003) 7:423–40. 10.1053/smrv.2002.0252 14573378

[40] Lachner-Piza D Jacobs J Bruder JC Schulze-Bonhage A Stieglitz T Dümpelmann M. Automatic detection of high-frequency-oscillations and their sub-groups co-occurring with interictal-epileptic-spikes. J Neural Eng. (2020) 17:016030. 10.1088/1741-2552/ab4560 31530748

[41] Pearce A Wulsin D Blanco JA Krieger A Litt B Stacey WC. Temporal changes of neocortical high-frequency oscillations in epilepsy. J Neurophysiol. (2013) 110:1167–79. 10.1152/jn.01009.2012 23761699 PMC3763087

[42] Piza DL Bruder JC Jacobs J Schulze-Bonhage A Stieglitz T Dumpelmann M. Differentiation of spindle associated hippocampal HFOs based on a correlation analysis. In: 38th Annual International Conference of the IEEE Engineering in Medicine and Biology Society (EMBC). (2016) p. 5501–4. 10.1109/EMBC.2016.7591972 28269503

[43] Helmstaedter C Lendt M Lux S. Verbaler Lern- und Merkfähigkeitstest: VLMT; Manual. Göttingen: Beltz Test. (2001).

[44] Rey. Rey L'examen Clinique en Psychologie. Paris Presses Universitaires de France. (1964).

[45] Helmstaedter C Grunwald Th Lehnertz K Gleißner U Elger CE. Differential involvement of left temporolateral and temporomesial structures in verbal declarative learning and memory: evidence from temporal lobe. Epilepsy Brain Cogn. (1997) 35:110–31. 10.1006/brcg.1997.0930 9339305

[46] Zentner J Wolf HK Helmstaedter C Grunwald T Aliashkevich AF Wiestler OD. Clinical relevance of amygdala sclerosis in temporal lobe epilepsy. J Neurosurg. (1999) 91:59–67. 10.3171/jns.1999.91.1.0059 10389881

[47] Gleissner U Helmstaedter C Schramm J Elger CE. Memory outcome after selective amygdalohippocampectomy in patients with temporal lobe epilepsy: one-year follow-up. Epilepsia. (2004) 45:960–2. 10.1111/j.0013-9580.2004.42203.x 15270763

[48] HELMSTAEDTER C. Eine modifizierte Version des Diagnostikums fur Cerebralschaden (DCS) zur Diagnostik raumlich-visueller Gedachtnisdefizite bei Patienten mit Temporallappenepilepsie. Epilepsie. (1991) 90.

[49] Gleiβner U Helmstaedter C Elger CE. Right hippocampal contribution to visual memory: a presurgical and postsurgical study in patients with temporal lobe epilepsy. J Neurol, Neurosurg Psychiat. (1998) 65:665–9. 10.1136/jnnp.65.5.665 9810934 PMC2170342

[50] Axmacher N Elger CE Fell J. Ripples in the medial temporal lobe are relevant for human memory consolidation. Brain. (2008) 131:1806–17. 10.1093/brain/awn103 18503077

[51] Lachner-Piza D Kunz L Brandt A Dümpelmann M Thomschewski A Schulze-Bonhage A. Effects of spatial memory processing on hippocampal ripples. Front Neurol. (2021) 12:237. 10.3389/fneur.2021.620670 33746877 PMC7973270

[52] Spencer SS. Neural networks in human epilepsy: evidence of and implications for treatment. Epilepsia. (2002) 43:219–27. 10.1046/j.1528-1157.2002.26901.x 11906505

[53] Bonilha L Rorden C Castellano G Pereira F Rio PA Cendes F. Voxel-based morphometry reveals gray matter network atrophy in refractory medial temporal lobe epilepsy. Arch Neurol. (2004) 61:1379–84. 10.1001/archneur.61.9.1379 15364683

[54] Bernhardt B Hong S-J Bernasconi A Bernasconi N. Imaging structural and functional brain networks in temporal lobe epilepsy. Front Hum Neurosci. (2013) 7:624. 10.3389/fnhum.2013.00624 24098281 PMC3787804

[55] McIntosh AM Kalnins RM Mitchell LA Fabinyi GCA Briellmann RS Berkovic SF. Temporal lobectomy: long-term seizure outcome, late recurrence and risks for seizure recurrence. Brain. (2004) 127:2018–30. 10.1093/brain/awh221 15215219

[56] Bernhardt BC Bernasconi N Concha L Bernasconi A. Cortical thickness analysis in temporal lobe epilepsy: Reproducibility and relation to outcome. Neurology. (2010) 74:1776–84. 10.1212/WNL.0b013e3181e0f80a 20513813

[57] Engel J McDermott MP Wiebe S Langfitt JT Stern JM Dewar S. Early surgical therapy for drug-resistant temporal lobe epilepsy: a randomized trial. JAMA. (2012) 307:922–30. 10.1001/jama.2012.220 22396514 PMC4821633

[58] Kennepohl S Sziklas V Garver KE Wagner DD Jones-Gotman M. Memory and the medial temporal lobe: Hemispheric specialization reconsidered. Neuroimage. (2007) 36:969–78. 10.1016/j.neuroimage.2007.03.049 17498975

[59] Witt J-A Coras R Schramm J Becker AJ Elger CE Blümcke I. The overall pathological status of the left hippocampus determines preoperative verbal memory performance in left mesial temporal lobe epilepsy. Hippocampus. (2014) 24:446–54. 10.1002/hipo.22238 24375772

[60] Kucewicz MT Saboo K Berry BM Kremen V Miller LR Khadjevand F. Human verbal memory encoding is hierarchically distributed in a continuous processing stream. eNeuro. (2019) 6. 10.1523/ENEURO.0214-18.2018 30847390 PMC6402539

[61] Kambara T Brown EC Silverstein BH Nakai Y Asano E. Neural dynamics of verbal working memory in auditory description naming. Sci Rep. (2018) 8:15868. 10.1038/s41598-018-33776-2 30367077 PMC6203730

[62] Clemens Z Mölle M Eross L Jakus R Rásonyi G Halász P. Fine-tuned coupling between human parahippocampal ripples and sleep spindles. Eur Journal of Neuroscience. (2011) 33:511–20. 10.1111/j.1460-9568.2010.07505.x 21138489

[63] Malow BA Carney PR Kushwaha R Bowes RJ. Hippocampal sleep spindles revisited: physiologic or epileptic activity? Clin Neurophysiol. (1999) 110:687–93. 10.1016/S1388-2457(99)00008-5 10378739

[64] Nobili L Baglietto MG Beelke M De Carli F De Negri E Rosadini G. Modulation of sleep interictal epileptiform discharges in partial epilepsy of childhood. Clin Neurophysiol. (1999) 110:839–45. 10.1016/S1388-2457(99)00021-8 10400197

[65] Frauscher B Bernasconi N Caldairou B von Ellenrieder N Bernasconi A Gotman J. Interictal hippocampal spiking influences the occurrence of hippocampal sleep spindles. Sleep. (2015) 38:1927–33. 10.5665/sleep.5242 26194569 PMC4667386

[66] Bruder JC Schmelzeisen C Lachner-Piza D Reinacher P Schulze-Bonhage A Jacobs J. Physiological ripples associated with sleep spindles can be identified in patients with refractory epilepsy beyond mesio-temporal structures. Front Neurol. (2021) 12:48. 10.3389/fneur.2021.612293 33643198 PMC7902925

[67] von Ellenrieder N Frauscher B Dubeau F Gotman J. Interaction with slow waves during sleep improves discrimination of physiologic and pathologic high-frequency oscillations (80–500 Hz). Epilepsia. (2016) 57:869–78. 10.1111/epi.13380 27184021

[68] Weiss SA Song I Leng M Pastore T Slezak D Waldman Z. Ripples have distinct spectral properties and phase-amplitude coupling with slow waves, but indistinct unit firing, in human epileptogenic hippocampus. Front Neurol. (2020) 11:174. 10.3389/fneur.2020.00174 32292384 PMC7118726

[69] Goyal A Miller J Qasim SE Watrous AJ Zhang H Stein JM. Functionally distinct high and low theta oscillations in the human hippocampus. Nat Commun. (2020) 11:2469. 10.1038/s41467-020-15670-6 32424312 PMC7235253

[70] Cimbalnik J Pail M Klimes P Travnicek V Roman R Vajcner A. Cognitive processing impacts high frequency intracranial eeg activity of human hippocampus in patients with pharmacoresistant focal epilepsy. Front Neurol. (2020) 11:1287. 10.3389/fneur.2020.578571 33193030 PMC7655124

