# Supplementary material for: Mesial-Temporal Epileptic Ripples Correlate With Verbal Memory Impairment
Source: Front Neurol. 2022 Jun 3;13:876024. doi: 10.3389/fneur.2022.876024 (PMC9204013; doi:10.3389/fneur.2022.876024)
Supplement: Supplementary file 1 [file Table_1.docx]

| **Patient** | **Age** | **Gender** | **Seizure type** | **MRI** | **surgery** | **SOZ** | **Implantation** |
| --- | --- | --- | --- | --- | --- | --- | --- |
| 1 | 12 | m | FAS, FIAS | tuberous sclerosis | R TL res & SAH | R mTL | R-HC, R-A, R-PHC, R-P, R-F |
| 2 | 54 | m | FIAS, FBTCS | L T-pole MEC, parenchymal lesions R F-bas & L-T-pole | L T-pole res | L mTL | L-HC, L-A, L-PHC |
| 3 | 33 | f | FAS, FIAS, FBTCS | no lesion | R tailored TL res | R TL w/o mTL | R-HC, R-A, R-PHC |
| 4 | 47 | m | FAS, FIAS | BL periventricular heterotopia, L HS | L SAH | L mTL | L-HC, L-A, L-PHC, L-O |
| 5 | 49 | m | FAS, FIAS | no lesion | R SAH | R mTL | L-HC, L-A, L-PHC, R-HC, R-A, R-PHC, R-F |
| 6 | 53 | m | FAS, FIAS, FBTCS | suspected FCD in R T-pole | R T-pole res & SAH | R mTL | R-HC, R-A, R-PHC, R-F |
| 7 | 27 | m | FAS, FBTCS | no lesion | R STG res | R STG | R-HC, R-A, R-PHC, R-F |
| 8 | 33 | m | FAS, FIAS, FBTCS | L mTL FCD | L post HC res & lesionectomy | L mTL | L-HC, L-A, L-PHC, L-F |
| 9 | 52 | f | FAS, FIAS, FBTCS | no lesion | L HC res & lesionectomy | L mTL | L-HC, L-A, L-PHC |
| 10 | 55 | f | FIAS, FBTCS | no lesion | L TL res & SAH | L mTL | L-HC, L-A, L-PHC, L-F, L-O |
| 11 | 60 | f | FAS, FIAS, FBTCS | BL T-pole MEC, L T-pole P lesion | L T-pole res | L T-pole | L-HC, L-A, L-PHC, R-PHC, R-A |
| 12 | 28 | f | FAS, FIAS | no lesion | L SAH | L mTL | L-HC, L-A, L-PHC |
| 13 | 23 | f | FAS, FIAS, FBTCS | R HS | R T-pole res & SAH | R & L mTL, T-pole | L-HC, L-A, L-PHC, R-HC, R-A, R-PHC |
| 14 | 23 | f | FAS, FIAS, FBTCS | no lesion | L SAH | L mTL | L-HC, L-A, L-PHC |
| 15 | 37 | f | FAS, FIAS, FBTCS | R T-pole MEC | R T-pole res | R T-pole | R-HC, R-A, R-PHC, R-T-pole |
| 16 | 34 | m | FAS, FIAS, FBTCS | L T-pole MEC | L T-pole res | L T-pole | L-HC, L-A, L-PHC |
| 17 | 12 | m | FAS, FIAS | suspected FCD in R T-pole | R TL res & SAH | R mTL | R-HC, R-A, R-PHC, R-F |
| 18 | 21 | f | FAS, FIAS, FBTCS | PCA WHO°I | R TL res & SAH | R mTL | R-HC, R-A, R-PHC, R-F |
| 19 | 34 | f | FAS, FIAS, FBTCS | ganglioglioma WHO°I | L FL/TL res & SAH | L FL, TL, mTL | L-HC, L-A, L-PHC, L-F, R-HC, R-A, R-PHC |
| 20 | 48 | m | FAS, FIAS, FBTCS | R F DVA | R O T res | R T O res | L-HC, L-A, L-PHC, R-HC, R-A, R-PHC, R-T, R-O |
| 21 | 31 | f | FAS, FIAS | BL HS | R TL res & SAH | R mTL | R-HC, R-A, R-PHC |
| 22 | 40 | f | FAS, FIAS, FBTCS | R HS | R TL res & SAH | R mTL | R-HC, R-A, R-PHC |
| 23 | 44 | f | FAS, FIAS, FBTCS | BL T P MEC | L T-pole res | L T-pole | L-HC, L-A, L-PHC, R-HC, R-A, R-PHC |
| 24 | 45 | m | FIAS, FBTCS | no temporal lesion | R TL res & SAH | R & L mTL | L-HC, L-A, L-PHC, R-HC, R-A, R-PHC |
| 25 | 22 | m | FAS | R HS | R SAH | R mTL | R-HC, R-A, R-PHC, R-F, R-P |
| **Abbreviations:** A = Amygdala; AC = arachnoid cyst; SAH = selective amygdalohippocampectomy; bas= basal; BL = bilateral; DVA: developmental venous anomaly; FL = frontal lobe; FAS = focal aware seizure; FBTCS = focal to bilateral tonic-clonic seizure; FCD = focal cortical dysplasia; FIAS = focal impaired awareness seizure; HC = hippocampal HS = hippocampal sclerosis; L = left; MEC = meningoencephalocele; mTL = mesial temporal lobe; O = occipital; P = parietal; PCA = pylocytic astrocytoma; PHC = parahippocampal; post = posterior; R = right; res= resection; STG = superior temporal gyrus; T = temporal; TL = temporal lobe | | | | | | | |
