# Supplementary material for: Mesial-Temporal Epileptic Ripples Correlate With Verbal Memory Impairment
Source: Front Neurol. 2022 Jun 3;13:876024. doi: 10.3389/fneur.2022.876024 (PMC9204013; doi:10.3389/fneur.2022.876024)
Supplement: Supplementary file 2 [file Table_2.docx]

# Table 2: Memory performances

| **Patient** | **Speech** | **IQ** | **Pre VM** | **Pre NVM** | **Post VM** | **Post NVM** | **Surgery** |
| --- | --- | --- | --- | --- | --- | --- | --- |
| 1 | L | N.A. | healthy | impaired | healthy | impaired | R TL incl MTL |
| 2 | L | 109 | healthy | impaired | healthy | healthy | L T-pole (MTL pres) |
| 3 | L | 104 | healthy | healthy | healthy | healthy | tailored TL res |
| 4 | L | 100 | impaired | impaired | impaired | healthy | L sAHE |
| 5 | L | 107 | healthy | healthy | healthy | healthy | R sAHE |
| 6 | L | N.A. | healthy | healthy | healthy | impaired | R T-pole res & AHE |
| 7 | L | 124 | healthy | healthy | healthy | healthy | R STG res |
| 8 | L | 94 | healthy | healthy | impaired | healthy | L post HC res & lesionectomy |
| 9 | L | 118 | healthy | impaired | impaired | impaired | L HC res & lesionectomy |
| 10 | L | 111 | impaired | impaired | impaired | impaired | L TL res & sAHE |
| 11 | L | 100 | healthy | impaired | impaired | N.A. | L T-pole res |
| 12 | L | 101 | healthy | healthy | impaired | impaired | L sAHE |
| 13 | L | 93 | healthy | impaired | healthy | impaired | R T-pole res & AHE |
| 14 | L | 92 | impaired | impaired | impaired | healthy | L sAHE |
| 15 | L | N.A. | healthy | healthy | healthy | healthy | R T-pole res |
| 16 | L | 95 | healthy | N.A. | healthy | healthy | L T-pole res |
| 17 | L | N.A. | healthy | healthy | healthy | healthy | R TL res & AHE |
| 18 | L | 121 | healthy | healthy | healthy | healthy | R TL res & AHE |
| 19 | L | 81 | impaired | impaired | impaired | impaired | R TL res & AHE |
| 20 | L | 143 | healthy | healthy | healthy | healthy | R O T res |
| 21 | L | 104 | healthy | impaired | healthy | impaired | R TL res & AHE |
| 22 | L | N.A. | healthy | impaired | healthy | healthy | R TL res & AHE |
| 23 | L | 93 | healthy | impaired | healthy | impaired | L T-pole res |
| 24 | L | N.A. | healthy | impaired | healthy | N.A. | R TL res & AHE |
| 25 | L | 97 | impaired | healthy | healthy | healthy | R sAHE |
| **Abbreviations:** L = left; rem mTL: remaining mesiotemporal structures (m)TL = (mesial) temporal lobe; N.A. = not available; NVM = Nonverbal Memory; Pre = pre-surgical; Post= post-surgical; VM= Verbal Memory;R= right | | | | | | | |
